# Alcohol, Aging, and the Stress Response

**Published:** 1999

**Authors:** Robert L. Spencer, Kent E. Hutchison

**Affiliations:** Robert L. Spencer, Ph.D., is an associate professor and Kent E. Hutchison, Ph.D., is an assistant professor in the Department of Psychology, University of Colorado at Boulder, in Boulder, Colorado

**Keywords:** aging, glucocorticoids, hypothalamic-pituitary axis, pituitary-adrenal axis, chronic AODE (effects of AOD [alcohol or other drug] use, abuse, and dependence), hormones, physiological stress, BAC (blood alcohol concentration), AOD tolerance, reinforcement, biological adaptation, literature review

## Abstract

The body responds to stress through a hormone system called the hypothalamic-pituitary-adrenal (HPA) axis. Stimulation of this system results in the secretion of stress hormones (i.e., glucocorticoids). Chronic excessive glucocorticoid secretion can have adverse health effects, such as Cushing’s syndrome. Alcohol intoxication activates the HPA axis and results in elevated glucocorticoid levels. Ironically, elevated levels of these stress hormones may contribute to alcohol’s pleasurable effects. With chronic alcohol consumption, however, tolerance may develop to alcohol’s HPA axis-activating effects. Chronic alcohol consumption, as well as chronic glucocorticoid exposure, can result in premature and/or exaggerated aging. Furthermore, the aging process affects a person’s sensitivity to alcohol and HPA axis function. Thus, a three-way interaction exists among alcohol consumption, HPA axis activity, and the aging process. The aging process may impair the HPA axis’ ability to adapt to chronic alcohol exposure. Furthermore, HPA axis activation may contribute to the premature or exaggerated aging associated with chronic alcohol consumption.

The fact that alcohol intoxication can relieve anxiety is well known. Paradoxically, those same intoxicating levels of alcohol also can induce excessive secretion of an important class of stress hormones, the glucocorticoids. (For a discussion of stress hormone production, see the sidebar entitled “ Regulation of Stress Hormone Production,” pp. 276–277.) Yet chronic alcohol exposure can trigger a tolerance to alcohol’s effects on the body’s stress response. For example, research has shown that healthy young rats can develop tolerance to alcohol’s stimulatory effects on glucocorticoid secretion—that is, the animals respond to chronic alcohol use by producing smaller increases in glucocorticoid levels. This same effect also appears to occur in humans. Research also indicates, however, that aged rats are much less able than younger rats^1^ to develop such tolerance (Spencer and McEwen 1997). Nonetheless, researchers do not know whether older humans likewise have a decreased ability to develop a tolerance to alcohol’s effects on stress hormones. Investigators do know, however, that chronic exposure in humans to both elevated glucocorticoid levels and alcohol produces symptoms resembling premature or exaggerated aging (Noonberg et al. 1985; Seeman and Robbins 1994).

**Regulation of Stress Hormone Production**

Humans generally are highly adept at confronting and fending off stress—which can be defined simply as any threat to a person’s physical and psychological well-being. Some of these threats are physical in nature, such as extreme temperature or extended lack of food or water. For most people, however, perceived threats to their well-being often are psychological in nature (e.g., work-related time pressures or stress from a relationship). Regardless of the source of the stress, the body responds by activating well-defined physiological systems that specialize in helping a person cope with the stress.

The two principal stress response systems in both humans and other animals are (1) a part of the nervous system called the sympathetic nervous system and (2) a hormone system called the hypothalamic-pituitary-adrenal (HPA) axis. Both systems enable the brain to communicate with the rest of the body. Activation of the sympathetic nervous system produces several physiological responses within seconds, such as an accelerated heart rate, increased respiration, and blood flow redistribution from the skin to the skeletal muscles. These responses facilitate the “fight or flight” behavioral response (Sapolsky 1993).

***The Role of the HPA Axis***

Activation of the HPA axis induces glucocorticoid secretion, which in turn affects a wide range of physiological responses, such as changes in blood sugar levels, and blood pressure, fat redistribution, muscle breakdown, and immune system modulation (Sapolsky 1993). Although these hormonal effects develop much more slowly (i.e., within hours) than those of the sympathetic nervous system, they may persist for several days and are vital to survival in the face of severe physical challenges. It is unclear, however, to what extent the activation of the sympathetic nervous system and the HPA axis helps combat psychological stress, which may not require such extensive physiological responses. The potential inappropriateness of HPA axis activation in the absence of physical stress is of special concern, because glucocorticoids exert such long-lasting effects.

The HPA axis consists of three groups of hormone-producing cells. They reside, respectively, in the brain region called the hypothalamus; in a hormone-secreting gland called the pituitary gland (located just below the hypothalamus); and in the adrenal glands, which are situated on top of the kidneys. These groups of cells act in a coordinated fashion to control the secretion of glucocorticoid hormones from the adrenal gland into general circulation.

Glucocorticoid secretion from the adrenal glands depends directly on the release of the adrenocorticotropic hormone (ACTH) from the pituitary gland and indirectly on the release of the corticotropin-releasing hormone (CRH) from the hypothalamus (see figure 2 in the article, p. 274). This hormone “cascade” becomes activated whenever CRH-producing nerve cells (i.e., neurons) in the hypothalamus are stimulated by neural input from other brain regions, usually in response to a stressful situation. As a result of this stimulation, these hypothalamic neurons secrete CRH into specific blood vessels located at the junction of the hypothalamus and the pituitary gland. CRH then is transported through these blood vessels to the pituitary gland (i.e., the anterior pituitary), where it stimulates specialized cells (i.e., corticotrope cells) to secrete ACTH into the bloodstream. Through the blood, ACTH is transported to the adrenal glands, where it induces certain cells to release glucocorticoids into the bloodstream. Thus, although glucocorticoid production and secretion occur in small glands located above the kidneys, these processes are ultimately controlled by the activity of various brain regions.

Glucocorticoid hormones have a wide range of regulatory effects on virtually every organ system in the body, including the central nervous system (i.e., the brain and spinal cord). Cortisol’s ability to affect many body systems allows this hormone to be an effective mediator of a generalized stress response. At the same time, however, the extensive range of cortisol’s effects necessitates tight regulation of the hormone’s levels. This control is achieved largely through a negative feedback mechanism (de Kloet et al. 1998). Thus, cortisol itself either directly or indirectly inhibits the CRH-producing neurons in the hypothalamus and the ACTH-producing cells in the anterior pituitary that control cortisol secretion, thereby blunting overall HPA axis activity and subsequent cortisol secretion.

Although this negative feedback normally is a highly effective means of ensuring that the body is not exposed to any more cortisol than is warranted by the conditions at hand, experimental evidence has indicated that with increasing age, this regulatory mechanism becomes impaired (Sapolsky et al. 1986). As discussed below, impaired cortisol negative feedback in older humans may contribute to a greater risk of alcohol-induced pathophysiology.

***Impairment of the HPA Axis and Its Consequences***

Researchers and clinicians have gained some insight into the consequences of extreme chronic elevation of cortisol levels from studying patients with Cushing’s syndrome, a disorder that is characterized by cortisol overproduction, usually caused by an adrenal or pituitary tumor. Symptoms include diabetes, muscle weakness, skin disorders, obesity of the torso, brittle bones (i.e., osteoporosis), disrupted menstruation, high blood pressure (i.e., hypertension), and increased susceptibility to infections (Veldman and Meinders 1996). Although chronic stress does not cause full-blown Cushing’s syndrome, the stress-induced chronic elevation of cortisol levels may exacerbate some related disorders, such as diabetes and osteoporosis, while simultaneously decreasing a person’s resistance to infectious agents.

Cushing’s syndrome is not the only disorder associated with abnormal cortisol levels. Dysregulation of cortisol secretion also occurs with some neuropsychological disorders, most notably depression, Alzheimer’s disease, chronic fatigue syndrome, posttraumatic stress disorder, and fibromyalgia (i.e., a recently defined syndrome of generalized pain) (de Kloet et al. 1998; Demitrack 1998). Researchers do not yet know, however, whether altered cortisol levels contribute to the development of these disorders or are merely a by-product of the conditions.

—Robert L. Spencer and Kent E. Hutchison

ReferencesDe KloetERVreugdenhilEOitzlMSJoelsMBrain corticosteroid receptor balance in health and diseaseEndocrinology Review19269301199810.1210/edrv.19.3.03319626555DemitrackMAChronic fatigue syndrome and fibromyalgiaPsychiatric Clinics of North America216716921998977480410.1016/s0193-953x(05)70031-9SapolskyRMNeuroendocrinology of the stress-responseBeckerJBBreedloveSMCrewsDBehavioral EndocrinologyCambridge, MAMIT Press1993287324SapolskyRMKreyLCMcEwenBSThe neuroendocrinology of stress and aging: The glucocorticoid cascade hypothesisEndocrine Reviews72843011986352768710.1210/edrv-7-3-284VeldmanRGMeindersAEOn the mechanism of alcohol-induced pseudo-Cushing’s syndromeEndocrine Reviews172622681996877135910.1210/edrv-17-3-262

This article examines the little-known, three-way relationship that exists among alcohol use and abuse, glucocorticoid secretion, and the aging process (see figure 1, p. 273). In particular, the article considers evidence that the glucocorticoid-based stress response system, as regulated by the hypothalamic-pituitary-adrenal (HPA) axis, plays a key role in the physiological and psychological responses to alcohol. The article also examines whether the stress hormone system contributes to age-related changes in a person’s response to alcohol (e.g., a reduced ability to develop tolerance to alcohol’s effects) and to alcohol-related changes in the aging process (e.g., nerve cell degeneration in some brain areas). By highlighting the overlap between these relationships, this article may spur further research on this important and complex topic.

## Alcohol’s Effects on HPA Axis Function in Young and Middle-Aged Individuals

### Alcohol-Induced Stimulation of the HPA Axis

**Figure 2:**
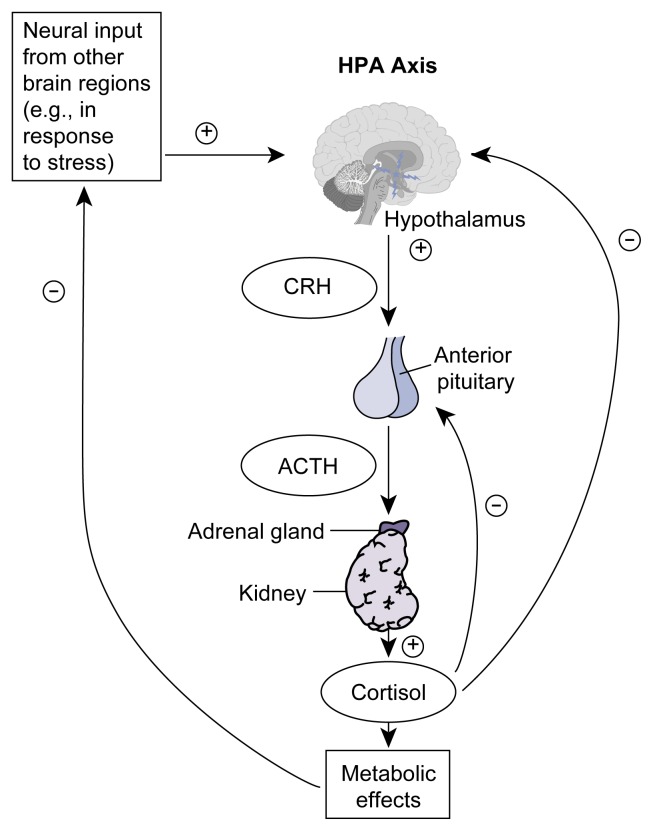
Schematic representation of the hypothalamic-pituitary-adrenal (HPA) axis. In response to stimulatory neural input from other brain regions (e.g., in stressful situations), certain cells in the brain’s hypothalamus secrete corticotropin-releasing hormone (CRH). This hormone stimulates cells in the pituitary gland, which is located below the hypothalamus, to secrete adrenocorticotropic hormone (ACTH) into the bloodstream. ACTH then is transported to the adrenal glands located atop the kidneys, where it activates certain cells to release cortisol, which exerts numerous metabolic effects. The HPA axis is regulated by both direct and indirect negative feedback mechanisms. Thus, cortisol directly inhibits further release of CRH from the hypothalamus and ACTH from the pituitary gland and indirectly lowers CRH secretion by reducing the neural input from other brain regions. NOTE: + = stimulatory effect; –= inhibitory effect.

Extensive documentation exists indicating that alcohol consumption reduces anxiety while it simultaneously activates the stress hormones through the HPA axis (see figure 2, also see sidebar entitled “ Regulation of Stress Hormone Production” ). In humans and other animals, the magnitude and duration of the glucocorticoid response depend on the amount of alcohol consumed (Spencer and McEwen 1990; Veldman and Meinders 1996). In response to alcohol, the levels of cortisol—the chief glucocorticoid hormone in humans—can be substantial and even surpass the levels typically seen in response to various stressful circumstances (Mendelson et al. 1971). Interestingly, blood alcohol concentrations (BACs) below 0.1 percent appear to have little effect on HPA axis activation (Jenkins and Connolly 1968). Furthermore, the 0.1 percent level has been (and in some States continues to be) considered a threshold for alcohol-related impairment and intoxication.

In addition to BACs, the extent to which alcohol leads to HPA axis activation appears to depend on genetic factors. Such a genetic influence is evident in people who have inherited a defective form of a particular gene that is involved in alcohol metabolism. Inheritance of this defective gene, which is especially prevalent among people of Asian descent, disallows the body to metabolize alcohol normally.^2^ People with the defective gene show significantly elevated blood cortisol levels, even at BACs below 0.1 percent (Wall et al. 1994). Other studies have found a greater HPA axis response to relatively low alcohol doses in people without family histories of alcoholism (Schuckit et al. 1996). This finding further supports the potential influence of genetic factors on the relationship between alcohol consumption and HPA activity.

The specific mechanism by which alcohol leads to HPA axis activation and elevated cortisol levels has not been conclusively established. One possibility is that alcohol disinhibits the HPA axis. In general, alcohol depresses nervous system activity. If some alcohol-sensitive nerve cells (i.e., neurons), in turn, exert inhibitory effects on the HPA axis, then the net effect of alcohol exposure would be HPA axis activation. A second possibility is that the HPA axis may be activated in response to certain stimulus properties of alcohol as part of a more coordinated, “whole body” stress response. Thus, a certain “ body wisdom” may recognize alcohol intoxication as stressful despite the concurrent reduced sense of anxiety.

### Tolerance to Alcohol’s Stimulatory Effects on the HPA Axis

People who repeatedly expose themselves to alcohol or other drugs develop, over time, tolerance to certain effects—in other words, these people experience lesser effects with the same dose or require higher doses to achieve the same effect. For example, tolerance develops to alcohol-related sedation, motor incoordination, and memory impairment (Poulos et al. 1981). Similarly, studies have shown that animals can develop tolerance to alcohol’s HPA axis-activating effects. For example, rats exposed to high alcohol doses daily for several weeks had an increase in the levels of corticosterone—the chief glucocorticoid hormone in animals—on day 14 that was only about one-half the increase observed on day 1 (Spencer and McEwen 1990). This tolerance development could not be explained by a change over time in their bodies’ ability to absorb or metabolize alcohol (i.e., development of metabolic tolerance), because the BACs achieved on day 14 were as high as those achieved on day 1 with the same alcohol dose.

However, researchers have not thoroughly studied the extent to which tolerance to alcohol’s stimulatory effects on the HPA axis develops in humans. In one study, alcohol administration to five alcoholics did not induce a significant increase in cortisol levels in the blood, even though the subjects’ BACs surpassed 0.1 percent (Merry and Marks 1969).^3^ Conversely, alcohol administration produced a substantial increase in cortisol levels in three of five purportedly nonalcoholic men in the study, suggesting that the alcoholics developed at least some degree of tolerance. (Interestingly, the two nonalcoholic men who did not show a significant cortisol response to alcohol on further questioning revealed an extensive history of recent alcohol use.)

Although some tolerance to alcohol’s effects on cortisol secretion may develop, it appears to be limited. For example, a study conducted in a controlled hospital setting found that men with a record of heavy and frequent alcohol use exhibited substantially elevated cortisol levels during an alcohol “ binge” (Mendelson et al. 1971). Furthermore, examination of seven different studies reveals that 6 to 40 percent of chronic alcohol users exhibited some of the symptoms of excessive cortisol production observed in Cushing’s syndrome (Veldman and Meinders 1996) (see textbox, p. 275). Thus, at least in some people, chronic alcohol use apparently can lead to chronically elevated cortisol levels with all the associated symptoms, a condition that sometimes has been called “pseudo-Cushing’s syndrome.”

**Cortisol’s Role and Effects in Humans**

The principal glucocorticoid hormone produced in humans in response to stressful experiences is cortisol. (Corticosterone is the primary stress hormone in rats and mice.)

Cortisol has potent effects, many of which help the body cope with various physical insults (e.g., adverse environmental conditions or injuries). Exposure to too much cortisol for too long, however, can have harmful consequences, including a condition called Cushing’s syndrome.

Because the majority of alcoholics do not develop pseudo-Cushing’s syndrome, most people probably experience some adaptation or tolerance to the HPA axis’ response to alcohol. This adaptation, however, may negatively affect the ability of the HPA axis and other physiological systems to maintain their normal functions (see table, p. 278). The cost of physiological adaptation to alcohol becomes evident when alcohol is suddenly withheld and the drinker experiences withdrawal symptoms (e.g., anxiety, increased heart rate, and tremors).

A few studies have shown that alcohol withdrawal also leads to excessive activation of the HPA axis (Mendelson et al. 1971; Adinoff et al. 1991). Thus, alcohol consumption, particularly in alcoholics, can interfere with normal HPA axis functioning during acute intoxication, chronic alcohol consumption (when tolerance development can alter HPA axis activity), and withdrawal after a bout of drinking. Moreover, the normal functioning of the HPA axis and its response to other stimuli appear to be compromised in alcoholics. In some studies various stimuli (e.g., surgical procedures or administration of the hormones insulin and ACTH [adrenocorticotropic hormone]) resulted in lower-than-expected HPA axis responses (Berman et al. 1990; Margraf et al. 1967). (For further discussion of ACTH and the HPA axis, see the sidebar entitled “ Regulation of Stress Hormone Production,” pp. 276–277.)

### Glucocorticoid Contributions to the Rewarding Effects of Alcohol

The interaction between alcohol and the HPA axis may be bidirectional—that is, not only does alcohol consumption stimulate cortisol secretion, but elevated cortisol levels may increase drinking by magnifying its rewarding effects. Evidence for the latter relationship between alcohol and the HPA axis derives from animal studies in which researchers experimentally manipulated corticosterone levels. In those studies, rats that produced no corticosterone because their adrenal glands had been surgically removed (i.e., the rats had been adrenalectomized) exhibited a dramatic reduction in voluntary alcohol intake (Fahlke et al. 1994). Treatment of the adrenalectomized rats with corticosterone restored their alcohol intake to levels comparable to, and sometimes higher than, the levels before the surgery. Similarly, rats with functional adrenal glands that were treated with a chemical preventing corticosterone production also exhibited decreased alcohol consumption (Fahlke et al. 1996). Finally, the injection of corticosterone directly into the brains of normal rats that displayed a moderate preference for alcohol resulted in enhanced alcohol intake in those animals (Fahlke et al. 1996).

Researchers have speculated that corticosterone may increase an individual’s alcohol consumption by enhancing alcohol’s rewarding effects (e.g., feelings of euphoria). The mechanism underlying this process may involve corticosterone-induced alterations in the levels of the brain chemical (i.e., neurotransmitter) dopamine in the brain region called the nucleus accumbens.^4^ In studies of the effects of cocaine or morphine exposure, corticosterone increased dopamine release in the nucleus accumbens (Piazza and Le Moal 1998), and that brain response appeared to be critical for the euphoria-inducing effects of the cocaine or morphine. Similarly, high glucocorticoid levels present in the brain during times of stress may facilitate alcohol’s rewarding properties, providing a potential explanation for survey results indicating that stress may contribute to heavy alcohol use in humans (see Fahlke et al. 1994). This aspect of the stress-alcohol interaction warrants further systematic investigation in humans.

Another variable that plays a role in the HPA axis’ response to both stress and alcohol’s effects is the aging process. Aging is associated with gradual, but often dramatic, changes over time in almost every physiological system in the human body. Combined, these changes result in decreased efficiency and resiliency of physiological function. The aging process is highly variable, however, with large individual differences in the overall rate of aging as well as in the specific patterns of age-related manifestations (Rowe and Kahn 1987).

As described in detail in the sidebar entitled “ Chronic Alcohol Consumption and Aging” (see pp. 281–283), considerable evidence suggests that a two-way interaction exists between alcohol abuse and aging. On the one hand, aging may alter a person’s physiological and psychological responses to alcohol. On the other hand, chronic alcohol use may alter the aging process, as indicated by several studies that found evidence for premature or exaggerated aging in chronic heavy drinkers (Evert and Oscar-Berman 1995; Noonberg et al. 1985; Pfefferbaum et al. 1992).

### Stress Hormone Activity and Aging

Just as a two-way interaction appears to exist between alcohol and aging—that is, (1) aging can modify the body’s response to alcohol, and (2) chronic alcohol exposure can modify the aging process—an analogous two-way interaction also appears to exist between stress and aging. This two-way interaction is probably mediated by the HPA axis. Thus, elderly people appear to have an impaired resiliency of the HPA axis response to the acute effects of stress. In addition, chronic stress may accelerate the aging process by causing overactivity of the HPA axis (Seeman and Robbins 1994).

### Age Differences in HPA Axis Function

Animal studies have demonstrated that HPA axis function changes as the animal enters the last quarter of its normal life span. Several studies have found that although rats of all ages experience a similar increase in HPA activity in response to stress, aged rats, compared with younger ones, show increased HPA activity in the absence of stress (i.e., increased basal activity) and a slower return to basal activity after a stress-induced increase in activity (see Sapolsky et al. 1986). Consequently, the bodies of aged rats are exposed to a substantially greater overall amount of glucocorticoid hormones than are the bodies of younger rats.

Studies in which rats were repeatedly exposed to the same stressful event also have found age-related changes in HPA axis function. In general, the HPA axis response decreases (i.e., habituation occurs) in response to repeated stress, similar to the tolerance development in response to repeated alcohol exposure.^5^ This habituation likely minimizes the amount of glucocorticoids to which the body is exposed. Aged rats, however, experience a slower rate of stress habituation than do younger rats (Spencer and McEwen 1997).

When analyzed together, the observed age-related changes in HPA activity suggest that the resiliency of the HPA axis in response to acute stress, as well as its ability to adapt to chronic stress, is impaired in older animals. Researchers have not yet identified the mechanism underlying this impaired HPA axis resiliency. One potential mechanism might involve changes in the levels of glucocorticoid receptors that contribute to the negative feedback regulation of glucocorticoid secretion, because aged rats tend to show reduced numbers of those receptors in certain brain regions (e.g., the hypothalamus and the hippocampus) (Sapolsky et al. 1986).

Researchers have not yet determined if impaired HPA axis resiliency also occurs during human aging. Older people, even those in their seventies, generally do not exhibit elevated basal cortisol levels (Seeman and Robbins 1994). However, researchers have not examined in elderly people the ability of the HPA axis to return to basal levels after acute stress or to habituate after repeated stress.

Some evidence indicates that elderly people may be less sensitive than younger people with respect to the negative feedback control of cortisol levels. For example, older people who received a dose of the potent synthetic glucocorticoid dexamethasone exhibited a blunted negative feedback response (Seeman and Robbins 1994) and thus were exposed to higher overall glucocorticoid levels than were younger people undergoing the same procedure. Furthermore, such an impaired response to dexamethasone occurs more consistently in older people suffering from major depression than in younger people with a similar degree of depression. Finally, older people suffering from Alzheimer’s disease or other forms of dementia also demonstrate a relatively high incidence of a blunted dexamethasone response (Seeman and Robbins 1994).

A series of studies have shown that dexamethasone exerts its primary negative feedback effects on the HPA axis by directly suppressing ACTH release from the pituitary (de Kloet et al. 1998). To date, researchers do not know the extent to which an impaired response to dexamethasone reflects a localized impairment in pituitary activity or changes in negative feedback sensitivity originating in other brain regions that affect pituitary function, such as the hypothalamus. On the other hand, cortisol is known to produce negative feedback effects on HPA axis activity by acting at the level of certain brain structures, such as the hypothalamus and hippocampus. A recent study showed impaired negative feedback sensitivity among older people (average age of 70) to cortisol (Wilkinson et al. 1997), suggesting that an age-related impaired sensitivity to glucocorticoid negative feedback is attributable to changes in the brain. Because negative feedback is an important mechanism that allows an organism to both recover from stress and turn off its HPA axis response to stress, impaired glucocorticoid negative feedback likely results in prolonged elevation of cortisol levels during stressful circumstances.

### Premature or Exaggerated Aging With Chronic Glucocorticoid Exposure

People generally believe that a “ hard life” (i.e., one fraught with difficulties, including repeated exposure to stressful situations) can lead to premature aging. Chronic stress-induced HPA axis overactivity may mediate such a process, a belief that is supported by research findings. For example, a study that examined post-mortem brains of vervet monkeys housed in a primate center detected extensive hippocampal damage in the brains of a subset of monkeys. These monkeys showed signs of chronic stress (e.g., gastric ulcers and bite scars) typically experienced by animals of low rank in the strict hierarchy found in this species (Uno et al. 1989). The hippocampus is vitally important for memory formation and, interestingly, has a high concentration of glucocorticoid receptors. Perhaps, as a result of the high glucocorticoid receptor levels, the growth and survival of many hippocampal neurons appear to depend on glucocorticoids (McEwen 1999). Some neurons, however, require the presence of only low glucocorticoid levels for survival (McEwen 1999) and may be damaged by high glucocorticoid levels. Thus, chronic high glucocorticoid exposure may cause the death of some hippocampal neurons and increase the susceptibility of other neurons to damage or death from other toxic insults, such as lack of oxygen (i.e., hypoxia).

During an organism’s lifetime, the effects of glucocorticoid exposure on brain cells may accumulate and contribute to neurodegeneration (Sapolsky et al. 1986). This hypothesis has been supported by studies in middle-aged rats whose adrenal glands were removed to prevent glucocorticoid production and who received lower-than-normal cortisol doses to replace the missing corticosterone. This treatment minimized the extent of hippocampal nerve cell loss that usually occurs with old age, although the treatment did not prevent an age-related cognitive decline in the animals (Landfield et al. 1981).

However, another study that assessed individual variations in the ability of aged rats to navigate a maze (a task that depends on hippocampal function) found a relationship between nerve cell degeneration and cognitive performance (Issa et al. 1990). Some aged rats performed significantly worse than did younger rats, whereas the performance of other aged rats did not differ from that of the younger ones. When the investigators examined the brains of the cognitively impaired aged rats, the animals exhibited significant nerve cell loss in the hippocampus compared with that of both the unimpaired aged rats and the younger rats.

To determine whether differences in glucocorticoid levels contributed to the group differences in performance and hippocampal degeneration, researchers screened the rats prior to death for their HPA axis response to acute stress. The analyses found that the corticosterone levels of the cognitively impaired aged rats took longer to return to basal levels after the end of the stressful event compared with younger or unimpaired aged rats. This observation is consistent with the belief that animals exposed to greater levels of glucocorticoids (e.g., as a result of HPA overactivity, such as prolonged corticosterone secretion in response to stress) exhibit signs of advanced aging in the brain, particularly in the hippocampus. It is important to point out, however, that the hippocampus also exerts a crucial inhibitory control over the HPA axis (Saplosky et al. 1986). Accordingly, age-related degeneration of the hippocampus could precede, rather than follow, HPA axis overactivity.

**Table:** Brain Areas Damaged by Chronic Alcohol Use and Their Functions

Brain Area	Function
Corpus callosum	Large structure extending from the front to the back of the forebrain that connects the right and left sides (i.e., hemispheres) of the brain
Frontal lobe	Contains the regions of the cortex devoted to the control of movement, language production, problem-solving ability, ability to formulate and execute plans, and control of appropriate social behavior
Hippocampus	A primitive area of the cortex that is vital for the formation of certain types of memories, especially memories of people, places, events, and factual information
Mammillary body	A subregion of the hypothalamus that contributes to the formation of certain types of memories and is frequently damaged in patients with a severe form of alcohol-induced memory deficits (i.e., Wernicke-Korsakoff syndrome)
Parietal lobe	Contains regions of the cortex devoted to the processing of the sense of touch, pain, and temperature; spatial processing; integrating movements with the surrounding world; mathematical ability; letter recognition; memory for nouns; and spatial memory
Temporal lobe	Contains regions of the cortex devoted to the processing of auditory information, language comprehension, categorization ability, and ability to identify objects

The hypothesis that chronic HPA axis overactivity may lead to hippocampal degeneration in humans has gained support from recent MRI studies indicating that people with depression (many of whom have elevated basal cortisol levels during depressive episodes) and patients with Cushing’s syndrome have, on average, reduced hippocampal volumes (Sapolsky 1996). In addition, the extent of hippocampal degeneration is correlated with the duration of depression or, in the case of Cushing’s syndrome, the severity of cortisol overproduction. Other studies, in which older nonhuman primates were exposed to chronic high cortisol levels, did not produce any detectable hippocampal degeneration (Levernz et al. 1999). Thus, high glucocorticoid levels alone may only increase the vulnerability of nerve cells to the harmful influences of additional factors. Such factors, associated with stress or pathological conditions, include low oxygen levels and elevated levels of potentially toxic molecules (e.g., free oxygen radicals and glutamate).

## Alcohol, the HPA Axis, and Aging—Research Implications

Few studies have examined the three-way interaction of alcohol, the HPA axis, and aging. As outlined in the sidebar “ Chronic Alcohol Consumption and Aging” (see pp. 281–283), aging likely alters the organism’s physiological and psychological responses to alcohol. Moreover, chronic alcohol abuse appears to exacerbate the aging process. The HPA axis and the aging body’s changing responses to glucocorticoids may serve as an important mediator of these processes. The existing evidence supporting this hypothesis is summarized in the following sections.

**Chronic Alcohol Consumption and Aging**

***Age-Related Changes in Alcohol Sensitivity***

Various factors may contribute to age-related differences in a person’s sensitivity to the effects of alcohol. For example, a given alcohol dose—even a single drink—can produce higher BACs in older people than in younger people. The main factor accounting for these higher BACs appears to be the increase in body fat relative to muscle that generally occurs with increasing age. Thus, compared with 25-year-olds, the percent of total body weight consisting of fat increases an average of 50 percent in 60-year-old women and an average of 100 percent in 60-year-old men (Dufour et al. 1992). Because alcohol dissolves only in water, of which muscle has a high content, but not in fat, the same alcohol dose results in a higher BAC in a person who has proportionately more fatty tissue and less body water. In addition, some evidence suggests that even with equivalent BACs, a given alcohol level has a greater impact on an older person’s physiological system than on that of a younger person (Atkinson 1990).

Like other physiological systems, the brain appears to experience an age-related increase in sensitivity to alcohol. For example, aged rats show an increased sensitivity to both the sedative and hypothermic effects of alcohol than do young adult rats (York 1983; Guthrie et al. 1987). Although to date no extensive studies on this issue have been conducted in humans, the older human brain also seems to be more sensitive to alcohol impairment of motor-coordination tasks (Vogel-Sprott and Barrett 1984).

Researchers do not yet know if age-related changes in sensitivity to alcohol affect a person’s susceptibility to developing alcohol abuse. People generally assume that alcohol abuse declines with age. However, several studies have noted that between 24 and 68 percent of alcohol-abusing people developed the first signs of alcohol abuse only after age 60 (Atkinson 1990). Interestingly, women and people of either sex with moderate to high socioeconomic status have shown the highest percentages of late-onset alcohol abuse (Atkinson 1990). These findings suggest that at least for some people, aging leads to an increased risk of alcohol abuse. Whether this increased risk results from age-related psychosocial factors (e.g., loss of a spouse, retirement, or loneliness) or changes in the physiological response to alcohol (or a combination of the two) remain to be determined.

***Alcohol Abuse and Premature or Exaggerated Aging***

Chronic alcohol exposure can lead to impairment of a wide range of physiological functions. Some of these pathological effects, such as alcohol-induced damage of liver and pancreas functions, appear to result directly from alcohol’s toxic effects on those organs. Other health problems associated with chronic alcohol use (e.g., cardiovascular disease, sleep disorders, gastrointestinal dysfunction, and increased susceptibility to infections), however, appear to be less specific in nature (Colsher and Wallace 1990; Brower et al. 1994). In fact, these effects, which vary from person to person, may represent an accelerated or exaggerated aging process.

Some researchers have noted that an important distinction may exist between alcohol-related accelerated aging versus exaggerated aging (Noonberg et al. 1985; Evert and Oscar-Berman 1995). Accelerated aging means that symptoms of old age appear earlier than normal, resulting in premature aging. Exaggerated aging implies that the symptoms of old age appear at the appropriate time, but in a more exaggerated form. Exaggerated aging may result from a person’s increased vulnerability to the pathophysiological changes that emerge during approximately the sixth decade of life, such as brittle bones (i.e., osteoporosis), adult onset diabetes, cognitive decline, and shrinkage (i.e., atrophy) of muscle tissue.

As discussed below, some evidence suggests that chronic alcohol exposure can lead to both accelerated and exaggerated aging. Nevertheless, some alcohol-induced pathological changes that superficially resemble the consequences of normal aging—and thus appear to indicate accelerated aging—have revealed, on closer inspection, some unique characteristics. For example, careful comparisons demonstrated that the nature of the memory deficits found in younger (i.e., mean age of 37.2 years) alcoholics differed from the deficits found in older (i.e., mean age of 64.7 years) nonalcoholics (Kramer et al. 1989).

Studies of the effects of long-term alcohol use on brain tissue and brain function have examined the connection between alcohol and the aging process. Advances in technologies to obtain brain images (i.e., neuroimaging technology) during the past 20 years have allowed researchers to study the brain structure of living individuals in great detail (i.e., with high spatial resolution). These studies have demonstrated that chronic alcohol use leads to substantial atrophy of the brain, as evidenced by reduced volumes of various brain regions (i.e., the cortex, anterior hippocampus, mammillary bodies, and corpus callosum). The brain regions most affected by chronic alcohol use appear to be the prefrontal and cerebellar cortex. The prefrontal cortex is the brain region believed to be most responsible for higher level cognitive processes, whereas the cerebellum plays an important role in motor function. Simultaneously, the volume of the fluid-filled cavities in the brain (i.e., the ventricles) increases—making up for lost tissue—after chronic alcohol use (Pfefferbaum et al. 1998).

Normal aging also appears to lead to a steady decline in the general cortical area (with the greatest decline occurring in the prefrontal cortex) and a concurrent enlargement of the ventricles. Nevertheless, several studies have demonstrated that these signs of cerebral atrophy are greater in alcoholics than in nonalcoholics of the same age (Pfefferbaum et al. 1992, 1998). Possibly, however, the alcoholics already possessed reduced cortical areas before the onset of alcohol abuse, and thus cortical atrophy may reflect a neuropathology that contributes to the susceptibility to alcohol abuse rather than a consequence of chronic alcohol exposure. To investigate this possibility, a recent study used magnetic resonance imaging (MRI) technology to examine changes in cortical area and ventricular size over a 5-year timespan in both alcoholics (average age of 45 at initial assessment) and nonalcoholics of similar age (average age of 51 at initial assessment) (Pfefferbaum et al. 1998). As in other studies, the cortical volume in the frontal, temporal, and parietal lobes was significantly reduced in the alcoholics compared with the nonalcoholics. In both groups, the cortical area declined during the 5-year study period. The alcoholics, however, exhibited a greater rate of decline in anterior superior temporal cortex volume than did the nonalcoholics, indicating that cortical atrophy likely represents a consequence of chronic alcohol use.

Another study found that the difference in cortical volume between alcoholics and nonalcoholics was greater among older subjects than among younger subjects (Pfefferbaum et al. 1992). In that study, after controlling for age, the extent of cortical atrophy in alcoholic men increased with age but not with duration of alcoholism. This observation suggests that the brains of older people may be more vulnerable to alcohol’s degenerative effects than the brains of younger people.

Consistent with the fact that chronic alcohol abuse can lead to reductions in cortical volume, numerous studies have observed alcohol-related deficits in cognitive functions. The most extreme cases of alcohol-related dementia and severe memory loss (i.e., amnesia), which constitute a condition called Wernicke-Korsakoff syndrome, may be primarily a result of severe alcohol-related nutritional and/or vitamin deficiencies (Nakada and Knight 1984). In addition, however, chronic alcohol abuse appears to produce more subtle—but significant—deficits in cognitive function. These deficits are most evident on tests of relatively complex cognitive function, such as the ability to follow abstract concepts or to adapt quickly to changing conditions (Tivis et al. 1995; Evert and Oscar-Berman 1995). On such tasks, the performance of alcoholics is impaired compared with nonalcoholics of the same age. In fact, chronic alcohol abuse appears to accelerate a person’s cognitive decline by approximately 10 years, because the cognitive performance of alcoholics generally is comparable to that of nonalcoholics who are 10 years older. This age-related discrepancy is apparent even in alcoholics in their thirties (Noonberg et al. 1985). Although these observations support the notion that alcoholics experience accelerated brain aging, the studies conducted to date have not ruled out the possibility that the alcohol-related cognitive deficits reflect an initial cognitive impairment that leads to an increased risk of alcoholism.

—Robert L. Spencer and Kent E. Hutchison

ReferencesAtkinsonRMAging and alcohol use disorders: Diagnostic issues in the elderlyInternational Psychogeriatrics255721990210129810.1017/s1041610290000308BrowerKJMuddSBlowFCYoungJPHillEMSeverity and treatment of alcohol withdrawal in elderly versus younger patientsAlcoholism: Clinical and Experimental Research18196201199410.1111/j.1530-0277.1994.tb00903.x8198220ColsherRLWallaceRBElderly men with histories of heavy drinking: Correlates and consequencesJournal of Studies on Alcohol515285351990227006110.15288/jsa.1990.51.528DufourMCArcherLGordisEAlcohol and the elderlyClinics in Geriatric Medicine812714119921576571EvertDLOscar-BermanMAlcohol-related cognitive impairments: An overview of how alcoholism may affect the workings of the brainAlcohol Health & Research World1989961995PMC687572731798082GuthrieSCooperRLThurmanRLinnoilaMPharmacodynamics and pharmacokinetics of ethanol, diazepam and pentobarbital in young and aged ratsPharmacology and Toxicology613083121987343822610.1111/j.1600-0773.1987.tb01825.xKramerJHBlusewiczMJPrestonKAThe premature aging hypothesis: Old before its time?Journal of Consulting and Clinical Psychology572572621989270861410.1037//0022-006x.57.2.257NakadaTKnightRTAlcohol and the central nervous systemMedical Clinics of North America681211311984631800010.1016/s0025-7125(16)31245-7NoonbergAGoldsteinGPageHAPremature aging in male alcoholics: “ Accelerated aging” or “ increased vulnerability”?Alcoholism: Clinical and Experimental Research9334338198510.1111/j.1530-0277.1985.tb05555.x3901803PfefferbaumALimKOZipurskyRBMathalonDHRosenbloomMJLaneBHaCNSullivanEVBrain gray and white matter volume loss accelerates with aging in chronic alcoholics: A quantitative MRI studyAlcoholism: Clinical and Experimental Research1610781089199210.1111/j.1530-0277.1992.tb00702.x1471762PfefferbaumASullivanEVRosenbloomMJMathalonDHLimKOA controlled study of cortical gray matter and ventricular changes in alcoholic men over a 5-year intervalArchives of General Psychiatry559059121998978356110.1001/archpsyc.55.10.905TivisRBeattyWWNixonSJParsonsOAPatterns of cognitive impairment among alcoholics: Are there subtypes?Alcoholism: Clinical and Experimental Research19496500199510.1111/j.1530-0277.1995.tb01537.x7625588Vogel-SprottMBarrettPAge, drinking habits and effects of alcoholJournal of Studies on Alcohol455175211984652147610.15288/jsa.1984.45.517YorkJLIncreased responsiveness to ethanol with advancing age in ratsPharmacology Biochemistry and Behavior19687691198310.1016/0091-3057(83)90346-56647504

### Age-Related Impaired Adaptation of the HPA Axis to Chronic Alcohol Exposure

One potential interaction among age, the HPA axis, and alcohol concerns alcohol’s stimulatory effect on the HPA axis, which research has suggested may be greater in old than in young or middle-aged people. To investigate this hypothesis, researchers have examined the changes in corticosterone levels in response to 14 daily alcohol treatments in both aged and younger rats (Spencer and McEwen 1997). In that study, both groups of animals displayed equivalent increases in corticosterone levels in response to alcohol on the first day of treatment, suggesting that in rats, no age-related difference exists in the initial response of the HPA axis to alcohol. In contrast, the ability of the HPA axis to adapt to repeated alcohol exposure differed greatly between the aged and the younger rats. Whereas young rats developed extensive tolerance in their corticosterone response to alcohol (i.e., showed smaller alcohol-induced increases in corticosterone levels) after 7 days of alcohol exposure, aged rats exhibited substantially less tolerance development. As a result, on days 7 and 14, the corticosterone levels in response to alcohol were significantly greater in the aged rats than in the younger ones. Such an age-related impairment in tolerance development to alcohol also has been observed in rats that had to perform a previously learned task while under the influence of alcohol (Mayfield et al. 1992).

Researchers have not yet determined the reason for impaired tolerance development in aged rats. Current investigations focus on the mechanisms that underlie tolerance development to alcohol in general. One potential mechanism involves an increased ability of the liver to break down (i.e., metabolize) and remove alcohol from the body after repeated alcohol exposure (i.e., metabolic tolerance). As a result, BACs resulting from a certain alcohol dose (and, by extension, alcohol’s effects on the body) would be lower after chronic alcohol consumption than after a onetime drinking episode. Although animal studies have shown that chronic, high-alcohol exposure increases the levels or functions of certain alcohol-metabolizing liver enzymes, this mechanism does not appear to be a major contributing factor to alcohol tolerance (Khanna et al. 1983). Similarly, no differences in BACs existed between the aged and younger rats on any of the test days in the study by Spencer and McEwen (1997).

Consequently, tolerance development to alcohol must result primarily from an overall decrease in the organism’s response to a given amount of alcohol after repeated alcohol consumption (i.e., functional tolerance). As indicated in the study by Spencer and McEwen (1997), this functional tolerance appears to develop more slowly in aged rats than in younger ones, providing some important clues to the aging process in general and to adaptation mechanisms to alcohol in particular. Researchers have examined several factors that may contribute to functional tolerance development, including direct changes in the cells’ response to alcohol, as well as changes in various systems that may help the body compensate for alcohol’s effects. Some of those physiological adaptations to repeated alcohol exposure involve “unconscious” learned responses that offset some of alcohol’s effects. For example, the body can “ learn” to increase body temperature in response to alcohol-related stimuli to offset alcohol-induced decreases in body temperature (i.e., alcohol’s hypothermic effects) (Mansfield and Cunningham 1980). Such complex mechanisms are especially vulnerable to the effects of aging and therefore are probably responsible for impaired alcohol tolerance development in older individuals. In addition, age-related changes at the cellular level may contribute to the body’s decreased ability to adapt to repeated alcohol exposure.

The impaired tolerance of the stress hormone response of aged rats to repeated alcohol exposure is reminiscent of the impaired habituation of the response of aged rats to repeated stress. Consequently, future studies must establish whether impaired alcohol tolerance reflects an age-related process specific to repeated alcohol exposure or to a consequence of aging that affects stress adaptation in general. Other investigations of the three-way interaction of alcohol, HPA activity, and aging should explore the possibility that greater cortisol responses to repeated alcohol exposure lead to an increase in alcohol’s rewarding effects in older people. Such an enhancement in alcohol’s rewarding effects could help explain the surprisingly high number of people who develop alcohol use disorders for the first time late in life (Atkinson 1990).

### Chronic HPA Activation, Chronic Alcohol Exposure, and Premature or Exaggerated Aging

To date, researchers have not investigated alcohol’s acute and chronic effects on HPA activity in elderly humans. However, many of the symptoms associated with excessive cortisol production in patients with Cushing’s syndrome (e.g., diabetes, muscle weakness, osteoporosis, atherosclerosis, hypertension, memory impairment, wasting away of brain tissue, sleep disturbances, and compromised immunity) also commonly occur in elderly people, especially among those who abuse alcohol. This overlap of the symptoms of aging and of chronic cortisol overexposure suggests that alcohol-induced excessive cortisol secretion is, at least in part, responsible for the premature or exaggerated aging seen in many alcoholics.

As described earlier, the chronic elevation of glucocorticoid levels may contribute to nerve cell degeneration in the hippocampus. Neuroimaging studies of the brains of living alcoholics, however, found significant reductions in the sizes of other brain areas in addition to the anterior hippocampus (Pfefferbaum et al. 1998). Although the hippocampus is structurally related to the cortex, glucocorticoids do not appear to have a degenerative effect on the cortex in general. Researchers must therefore determine whether elevated cortisol levels contribute to a general alcohol-induced neurodegeneration or account only for hippocampal degeneration. Even a selective cortisol-mediated alcohol effect on the hippocampus is of serious concern because of the central role of that brain region in memory processes.

## Conclusions and Treatment Implications

The findings reviewed in this article suggest that numerous interactions exist among chronic alcohol consumption, HPA activity, and the aging process. For example, alcohol-related overactivity of the HPA axis and the resulting elevated cortisol levels may contribute to premature or exaggerated aging in many people with a long history of alcohol abuse. In addition, elderly people may be more susceptible than younger people to a stress-induced pattern of drinking, because alcohol elicits greater cortisol responses that may enhance alcohol’s rewarding properties. Finally, elevated cortisol responses in older people may intensify the pathophysiology associated with alcohol abuse, even in people who develop alcohol use disorders late in life.

Based on these observations, some researchers have speculated that therapeutic approaches to inhibit cortisol secretion and/or cortisol’s effects might be useful as a potential adjunct to alcoholism treatment. Because cortisol is such an important hormone that normally helps regulate many physiological processes in the body, medications that completely suppress its production or activity would not likely be a viable treatment for chronic alcoholics. Nevertheless, as researchers gain a better understanding of the regulation of the HPA axis, they may discover treatments to effectively modulate HPA axis activity and minimize the development of HPA axis overactivity. Such treatments may be useful in reducing the pathophysiology associated with chronic alcohol abuse and may reduce alcohol’s rewarding effects, which contribute to its addictive nature.

## Figures and Tables

**Figure 1:**
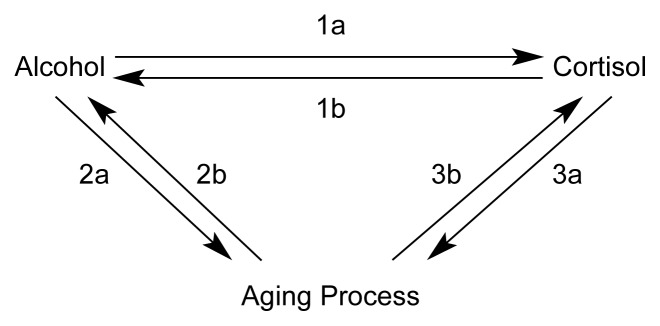
The three-way interaction of alcohol, cortisol secretion, and the aging process. Cortisol secretion is an indicator of the activity of the hypothalamic-pituitary-adrenal (HPA) axis, a hormone system that coordinates the stress response. Alcohol consumption stimulates cortisol secretion (1a). In turn, cortisol facilitates alcohol’s rewarding effects (1b). Chronic alcohol consumption also can lead to premature and/or exaggerated aging (2a). Conversely, the aging process results in increased blood alcohol levels following consumption of the same alcohol dose as well as increased vulnerability to alcohol’s effects, including alcohol’s abuse potential (2b). Finally, chronic cortisol elevation also results in premature and/or exaggerated aging (3a), and the aging process can lead to increased cortisol secretion by impairing the organism’s ability to adapt to stress (3b).

## Footnotes

1 The typical life span of a rat is 2 to 2.5 years. In general, young adult rats are 4 to 5 months old, whereas aged, or “older,” rats are 22 to 24 months old.

2 The inability to normally metabolize alcohol can result in unpleasant symptoms in response to alcohol consumption, including visible flushing of the face, nausea, and vomiting.

3 Studies such as these, in which alcohol is administered to alcoholics, are no longer conducted in human subjects.

4 This brain region appears to be essential for the rewarding or euphoria-inducing effects of alcohol or other drugs and serves as a link between brain regions that generate emotions and brain regions that control movement.

5 The terms “tolerance” and “habituation” both refer to the same phenomenon—the diminished response to a certain stimulus. However, by convention, the term “tolerance” is used for a diminishing response to drugs, whereas the term “habituation” generally is used with nondrug stimuli, such as stress or repeated sounds.
